# Supplementary material for: The Spanish version of the Fatigue Assessment Scale: reliability and validity assessment in postpartum women
Source: PeerJ. 2017 Sep 27;5:e3832. doi: 10.7717/peerj.3832 (PMC5622603; doi:10.7717/peerj.3832)
Supplement: Supplemental Information 1 [file peerj-05-3832-s001.pdf]

FATIGUE ASSESMENT SCALE – English translation of the Spanish Version (FAS-e)

Authors: Cano-Climent, Antoni; Oliver-Roig, Antonio Cabrero-García, Julio; De Viers, Jolanda; Richart-Martínez, Miguel.

Instructions:

The next ten sentences are about how you usually feel. For each one of them mark one of the five possible answers.

1. I am worried about fatigue.

1. Never 2. Sometimes 3. Normally 4. Very often 5. Always

2. I get tired very quickly.

1. Never 2. Sometimes 3. Normally 4. Very often 5. Always

3. I don't do much during the day.

1. Never 2. Sometimes 3. Normally 4. Very often 5. Always

4. I have enough energy for everyday life.

1. Never 2. Sometimes 3. Normally 4. Very often 5. Always

5. Physically, I feel exhausted.

1. Never 2. Sometimes 3. Normally 4. Very often 5. Always

6.I have problems starting things.

1. Never 2. Sometimes 3. Normally 4. Very often 5. Always

7. I have problems thinking clearly.

1. Never 2. Sometimes 3. Normally 4. Very often 5. Always

8. I feel no desire to do anything.

1. Never 2. Sometimes 3. Normally 4. Very often 5. Always

9. Mentally, I feel exhausted.

1. Never 2. Sometimes 3. Normally 4. Very often 5. Always

10. When I am doing something, I can concentrate quite well.

1. Never 2. Sometimes 3. Normally 4. Very often 5. Always

FATIGUE ASSESMENT SCALE – Spanish Version (FAS-e)

Authors: Cano-Climent, Antoni; Oliver-Roig, Antonio Cabrero-García, Julio; De Viers, Jolanda; Richart-Martínez, Miguel.

Instrucciones:

Las siguientes diez frases se refieren a cómo te sientes habitualmente. Para cada una de ellas marca una de las cinco posibles respuestas.

1. Estoy preocupada/o por la fatiga

1. Nunca 2. A veces 3. Normalmente 4. Muy a menudo 5. Siempre

2. Me canso muy rápidamente

1. Nunca 2. A veces 3. Normalmente 4. Muy a menudo 5. Siempre

3. No hago mucho durante el día.

1. Nunca 2. A veces 3. Normalmente 4. Muy a menudo 5. Siempre

4.Tengo bastante energía para el día a día

1. Nunca 2. A veces 3. Normalmente 4. Muy a menudo 5. Siempre

5. Físicamente, me siento agotada/o.

1. Nunca 2. A veces 3. Normalmente 4. Muy a menudo 5. Siempre

6.Tengo problemas para empezar cosas

1. Nunca 2. A veces 3. Normalmente 4. Muy a menudo 5. Siempre

7. Tengo problemas para pensar claramente

1. Nunca 2. A veces 3. Normalmente 4. Muy a menudo 5. Siempre

8. No tengo ganas de hacer nada

1. Nunca 2. A veces 3. Normalmente 4. Muy a menudo 5. Siempre

9. Mentalmente, me siento agotada/o

1. Nunca 2. A veces 3. Normalmente 4. Muy a menudo 5. Siempre

10.Cuando hago algo, puedo concentrarme bastante bien

1. Nunca 2. A veces 3. Normalmente 4. Muy a menudo 5. Siempre
